# Divergent selection on behavioural and chemical traits between reproductively isolated populations of *Drosophila melanogaster*

**DOI:** 10.1111/jeb.14007

**Published:** 2022-04-12

**Authors:** Bozhou Jin, Daniel A. Barbash, Dean M. Castillo

**Affiliations:** ^1^ Department of Molecular Biology and Genetics Cornell University Ithaca New York USA; ^2^ 5922 Department of Biology University of Nebraska at Omaha Omaha Nebraska USA

**Keywords:** mate choice, natural selection, sexual selection, speciation

## Abstract

Speciation is driven by traits that can act to prevent mating between nascent lineages, including male courtship and female preference for male traits. Mating barriers involving these traits evolve quickly because there is strong selection on males and females to maximize reproductive success, and the tight co‐evolution of mating interactions can lead to rapid diversification of sexual behaviour. Populations of *Drosophila melanogaster* show strong asymmetrical reproductive isolation that is correlated with geographic origin. Using strains that capture natural variation in mating traits, we ask two key questions: which specific male traits are females selecting, and are these traits under divergent sexual selection? These questions have proven extremely challenging to answer, because even in closely related lineages males often differ in multiple traits related to mating behaviour. We address these questions by estimating selection gradients for male courtship and cuticular hydrocarbons for two different female genotypes. We identify specific behaviours and particular cuticular hydrocarbons that are under divergent sexual selection and could potentially contribute to premating reproductive isolation. Additionally, we report that a subset of these traits are plastic; males adjust these traits based on the identity of the female genotype they interact with. These results suggest that even when male courtship is not fixed between lineages, ongoing selection can act on traits that are important for reproductive isolation.

## Graphical abstract

Selection acts on male courtship traits in different directions in African and non‐African populations of *D.* *melanogaster*. This divergent selection leads to the maintenance of different courtship traits in these populations. Selection on some traits, such as signing, could also lead to phenotypic plasticity, where males adjust their behavior based on the female genotype they are courting. Together these observations suggest a mechanism for how divergent selection and plasticity can contribute to asymmetrical premating isolation between these populations.

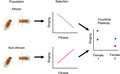

## 1 INTRODUCTION

Sexual selection is a powerful force that drives courtship behaviour evolution in populations, and can eventually lead to reproductive isolation (Andersson, 1994; Boughman, 2002; Coyne & Orr, 2004; Kirkpatrick, 1982; Ritchie, 2007). This observation is supported by comparative studies that quantify rapid trait evolution across widely diverged clades (Barraclough et al., 1995; Moller & Cuervo, 1998) as well as by studies that estimate selection gradients on sexual traits in focal populations within species (Brooks & Endler, 2001; Callander et al., 2012; Hill, 1991; Oh & Shaw, 2013; Rebarm et al., 2009; Steiger & Stokl, 2014). However, sexual selection alone might be insufficient to maintain species differences in the face of gene flow (Servedio & Bürger, 2014). Sexual selection can vary between populations, which could be a prerequisite for divergent selection and speciation (Chenoweth et al., 2010; Watts et al., 2019), but documenting ongoing divergent selection in the context of lineage divergence is more difficult (Higgie et al., 2000; Langerhans & Makowicz, 2013; Maan et al., 2004, 2010; Pauers & Mckinnon, 2012; Selz et al., 2016; Svensson et al., 2006; Wilkins et al., 2016). The difficulty in making connections between ongoing sexual selection and premating reproductive isolation may reflect that the most intensely studied traits for premating reproductive isolation are conspicuous fixed differences between fully isolated species (Coyne & Orr, 2004; Mckinnon & Rundle, 2002; Qvarnstrom et al., 2010). This contrasts with recently diverged species that may not show fixed differences in key mating‐related traits, but nevertheless show strong reproductive isolation (Hendry et al., 2009; Khallaf, Auer, et al., 2020; Mallet, 2008; Merot et al., 2017). By estimating selection gradients in lineages that experience gene flow yet show strong isolation, one can identify traits might contribute to reproductive isolation and whether ongoing selection reflects population level divergence.

One reason why it is important to study ongoing (i.e., contemporary) sexual selection is because the targets or intensity of sexual selection may have changed over the course of speciation (Price, 1998; Schluter & Price, 1993), especially if mating signals are context‐ or environment‐dependent (reviewed in Candolin, 2019). When species are fully reproductively isolated, ongoing sexual selection can continue to act, but on mating traits not directly associated with reproductive isolation (Boake et al., 1997; Ryan & Rand, 1993). In addition, divergent traits may indirectly contribute to reproductive isolation, or maybe have diverged from selection from the abiotic environment, but now function in sexual isolation (Coyne & Orr, 2004; Ritchie, 2007). In nascent lineages, however, one is much more likely to identify divergent selection on traits that contribute directly to reproductive isolation. One challenge for studying nascent lineages is that suites of traits show correlated divergence (Hohenlohe & Arnold, 2010; Oh & Shaw, 2013), which makes it difficult to disentangle which traits are important for female mate choice (Hohenlohe & Arnold, 2010). This can be especially true when there are overlapping trait values between lineages, or if males have the potential to change their courtship based on female identity (Berdan et al., 2019; Fox et al., 2019; Pfennig et al., 2010).

In models of speciation by sexual selection, it is often assumed that there are single optimal male traits that are selected by a uniform female preference (Kirkpatrick, 1982; Lande, 1981). However, variation in female preference often occurs, where one male phenotype is not uniformly preferred (Jennions & Petrie, 1997; Kelley, 2018; Mendelson et al., 2014; Rebar & Rodriguez, 2013). Frequency‐dependent selection can lead to the maintenance of variation in male courtship traits and female preferences (Otto et al., 2008), which, in turn, can lead to rapid evolution of reproductive isolation if populations become geographically isolated (Castillo & Delph, 2016; Mendelson et al., 2014; Otto et al., 2008). Variation in female mate preference could also maintain selection for male courtship plasticity, if males maximize fitness by tailoring courtship to match female preference. Males can alter mating‐related traits such as the intensity of courtship or size of ejaculate, based on the presence of rivals or on the mating status of females (reviewed in Bretman et al., 2011; Kelley & Jennions, 2011; Otte et al., 2018; Petfield et al., 2005). Additionally, plasticity caused by imprinting and social environment can change mating traits that facilitate reproductive isolation (Li et al., 2018; Marie‐Orleach et al., 2019, 2020; Yang et al., 2019). However, the potential for male behavioural plasticity in response to female cues during courtship as it relates to speciation has not been investigated.

In this study we use strains of *Drosophila melanogaster* representing populations that show strong asymmetrical reproductive isolation to determine which traits are under divergent selection. *Drosophila* *melanogaster* originated in southern Africa and migrated out of Africa in the past 10 000–15 000 years (Kapopoulou et al., 2018; Li & Stephan, 2006; Pool et al., 2012). Early reports documented behavioural isolation between strains collected in Southern Africa and strains collected outside of Africa (Hollocher et al., 1997; Wu et al., 1995). The Southern African strains were regarded as a single lineage that remained in the ancestral range, called Z‐type, and non‐African strains composed the cosmopolitan lineage and were called M‐type. Reproductive isolation between these lineages is asymmetric; M‐type females mate indiscriminately when given a choice between males of each type, while Z‐type females typically show strong preference for Z‐type males (Hollocher et al., 1997; Wu et al., 1995). While strong reproductive isolation persists between these strains of *D*. *melanogaster*, we now understand that the relationship between Z‐type and M‐type lineages is more complicated. For example, there is not a single genetic lineage in southern Africa that corresponds to Z‐type, instead the behaviour of female rejection of non‐African males, is spread across populations and lineages (Coughlan et al., 2021). In contrast, out‐of‐Africa strains are more homogenous, likely representing a bottleneck from their migration event (Kapopoulou et al., 2018; Li & Stephan, 2006). In addition, populations that have admixed ancestry show distinct patterns of reproductive isolation when tested against African and North America strains (Yukilevich & True, 2008). This suggests that different behavioural traits and preferences might also segregate within regions of Africa.

Given that female preference has evolved rapidly between populations, it is assumed that male courtship has also rapidly evolved. While many traits have been documented to be divergent between populations, initially in the context of Z/M‐type lineages (Grillet et al., 2012; Moran, 2006), it remains necessary to understand whether selection is currently operating on any of these traits. To answer this question, we have here estimated selection gradients on courtship behaviours and cuticular hydrocarbons for representative non‐African and Southern African female genotypes to determine if divergent selection is acting on specific traits. From previous work using common lab type strains, we could make *a priori* predictions about the traits that might be under positive selection in non‐African strains (Grillet et al., 2006; Kurtovic et al., 2007; Scott et al., 2011; Talyn & Dowse, 2004; Wu et al., 1995), but identifying traits under selection in the Southern African strain is an essential step to eventually connect sexual selection to reproductive isolation. Cuticular hydrocarbons have been implicated in reproductive isolation between populations through the use of CHC transfer experiments, where entire pheromone repertoires are transferred between individuals (Coyne et al., 1999; Grillet et al., 2012). Thus it is important to understand which specific CHCs are under selection. Leveraging diverse genotypes from Southern Africa, we identified several behavioural traits and cuticular hydrocarbons under divergent selection or female strain‐specific selection. Some of these traits were plastic and female‐genotype‐dependent, which may influence the evolution of divergent signals. Overall we identified male signalling traits that are strong candidates for being under contemporary divergent selection in *D*. *melanogaster* populations and showed that they are possibly contributing to reproductive isolation.

## 2 METHODS

### 2.1 Fly stocks

All stocks were reared on standard yeast‐glucose media prepared and kept at room temperature (~22°C). These stocks were chosen to maximize variation in courtship and cuticular hydrocarbon phenotypes that are observed in *D*. *melanogaster*. For all mating experiments and cuticular hydrocarbon extractions, virgin males and females were collected and isolated until they were 7–10 days old. While *D*. *melanogaster* are capable of mating 8 h after they eclose, and generally are fully mature by 3–5 days, we found that the Southern African flies produced more robust courtship after age of 7–10 days. Each male fly was kept in an individual vial since male mating behaviour is affected by their interactions with other males (Dixon et al., 2003).

The non‐African *D*. *melanogaster* strains used were Canton‐S (line maintained by Mariana Wolfner) and DGRP‐882 (BDSC #28255). For a non‐African female strain we wanted to use a strain that had little or no admixture with African populations. Admixture has been estimated for individuals of the DGRP panel (Pool, 2015) and DGRP‐882 has among the least amount. Canton‐S was used because it a strain commonly used in behavioural experiments. The remaining lines were chosen to maximize diversity in courtship and cuticular hydrocarbon phenotypes, so we focused on strains collected in Southern Africa. One set of strains was collected in the mid‐1990s and has been assayed for female mate preference but not male behaviour (Hollocher et al., 1997; Wu et al., 1995). The Z in the strain name reflects the collection location of Zimbabwe but not necessarily the female mating behaviour or ‘Z‐type’ status. These include strains Z53 and Z30 (provided by Trudy Mackay), strain Z29 (BDSC #60741), strain ZS11 (provided by Chip Aquadro), and strains ZH33 and ZH42 (provided by Andy Clark; Grenier et al., 2015). Strains ZK82 and ZK58 were collected in Lake Kariba, Zimbabwe but not previously phenotyped for male or female behaviour (provided by Chip Aquadro). The strains Lower Zambize 2.1 (LZ21), Chipata 11 (CH11), Chipata 12 (CH12), Livingstone 4.7 (LS47), and Lusaka Camp 4 (LC4) were collected in Zambia in 2015 by Daniel Matute. Strain collection date did not predict male courtship or CHC phenotype as strains from different collection times clustered in a principal component analysis of courtship behaviour and CHC phenotype (see Section 3). For assaying Southern Africa females we chose Z53 because it has well documented female behaviour that has been consistent since its initial collection (Moran, 2006; Wu et al., 1995). The *Drosophila simulans* strain that we used as a reference for scissoring behaviour, SA22, was provided by Chip Aquadro (originally collected in Stellenbosch South Africa).

### 2.2 Video recording and analysis of courtship behaviour

To record multiple courtship interactions simultaneously, we used a chamber design that allowed for easy loading of individuals with a mechanism to keep individuals separate until recording started (Koemans et al., 2017). Pairs of individuals were also isolated from all other courting pairs. All mating experiments were carried out within 2 h of lights on (between 9:00 AM and 11:00 AM local time) as this is the time we could observe the maximum number of copulations. In pilot recordings we found no differences in courtship intensity or copulation rate for recordings that were done early or late in our recording window. The recording chamber was placed into a lightbox with LED strips (Konseen 16" Square Mini Dimmable Photo Light Box) to control light intensity across trials and courtship was recorded for 30 min with a camera (RoHS 0.3MP B&W Firefly MV USB 2.0 Camera). We used two courtship chambers to allow each chamber to be empty between recordings so any volatile cues from the flies would dissipate.

We used the software BORIS (Friard & Gamba, 2016) to quantify male behaviour. Behaviours were classified manually because we were interested in potentially identifying novel behaviours. We recorded all behaviours as state behaviours at 10 s intervals (similar to Gaertner et al., 2015). This might underestimate the time spent on a behaviour, but still captures large differences in behavioural sequence since interactions were 10.5 min long on average.

During the first stage of observations, we attempted to identify all behaviours that were occurring. Most were well described elements including singing, chasing, licking, attempted‐copulation, copulation, and scissoring (Cobb et al., 1985; Cobb & Jallon, 1990). We noticed that singing behaviour occurred at two positions relative to the female, so we designated a separate singing (singing‐2 is mostly from in front of the female, head‐to‐head, compared to ‘normal’ singing with the male behind or slightly to the side of the female). We also noticed several ‘circling’ behaviours where the male moved in an arc around the female while wing displaying (singing or scissoring; Video S1). The frequency of circling behaviours across this initial dataset indicated that only one circling behaviour was frequent enough to reliably identify, so all circling behaviours were combined into a single metric. After watching many videos we could not reliably score licking and tapping, so we combined these with chasing and following behaviours into ‘engaging’. This left eight categories: separate, engaging, singing, singing‐2, scissoring, circling, attempted‐copulation, and copulating.

When scoring the male behaviours, the video was run at 2× speed and paused every 10 s to record a behaviour. The observer recorded observations blindly with respect to genotype. The scoring for trials that successfully copulated were ended as soon as the flies started copulation. There were several trials that were unsuccessful in mating that we watched to make specific comparisons (see below in Section 2.6). These unsuccessful trials were quantified for a time equal to the average copulation latency of the successful trials for the same genotype combinations.

### 2.3 Cataloguing potential African male behaviours

Though the courtship sequence of *D*. *melanogaster* is well known, this information comes primarily from non‐African lab strains (Cobb et al., 1985) so it was critical to establish which, if any, additional behaviours may occur in African strains. A comparison of the time spent on these stereotypical courtship traits identified differences between African and non‐African males (Moran, 2006), but it was unclear if additional behaviours were observed or quantified. We used the *D*. *melanogaster* genotypes Z53 and DGRP‐882 in this initial classification since we could compare their behaviours to previous reports (Gaertner et al., 2015; Moran, 2006). *Drosophila* *simulans* served as a standard for the scissoring behaviour as it is a frequent part of their courtship display (Cobb et al., 1985; Video S2) and some genotypes of *D*. *melanogaster* with African ancestry may engage in this behaviour (Yukilevich & True, 2008). We recorded six pairs of flies during every trial block. This balanced efficiency with the need to record pairs at a close distance to document subtle behaviours.

### 2.4 Selection acting on courtship behaviours

Our second stage of recording was aimed at identifying behaviours under selection by correlating mating behaviours with copulation success and copulation latency, which was our proxy for mating success and fitness (Hoffmann, 1999; Taylor et al., 2007). We conducted trials between Z53 or DGRP‐882 females and males from 15 strains that made up our panel. During each block, 10 pairs of flies were video recorded simultaneously with five pairs courting Z53 females and the remaining five courting DGRP‐882 females. We used five male strains per block such that the Z53 and DGRP‐882 females were paired with the same strains within a block, with the male strain determined from a randomized sequence of five. The order of the sequence was rotated after every three blocks of recording, so that each strain had equal probability of being in an early versus late block across the course of the experiments. This randomization scheme limited the effects on courtship intensity of time of day and of blocks occurring across multiple weeks. We repeated this scheme until almost all female × male strain combinations had five successful mattings, which resulted in some combinations having more successful mattings than others. Some specific genotype combinations did not mate after multiple attempts including Z53 females with DGRP‐882, Canton‐S, and LZ21 males and DGRP‐882 females with Z30 and LZ21 males. Since LZ21 males did not mate with either female strain, we did not include them in any selection analyses.

### 2.5 Cuticular hydrocarbon collection and quantification

We collected cuticular hydrocarbons (CHCs) from three males and three females for all of the *D*. *melanogaster* strains used in the behavioural studies. CHCs are influenced by both temperature and humidity so we ensured that all collections were done in parallel to the behavioural observations (Noorman & Den Otter, 2002; Savarit & Ferveur, 2002). Male and female virgins were collected, stored individually and were 7 days old before CHCs were extracted in 8 dram glass vials with 100 µl HPLC grade hexane (99% purity) for 1 min on an orbital shaker. The solvent was then transferred into an autosampler vial with a small volume insert, left in a fume hood for 4 h to evaporate, and vials sealed and placed at −20°C until analysis. For analysis CHCs were re‐eluted in 50 µl hexane with an internal standard (hexacosane C26) based on Dembeck et al. (2015).

To measure plasticity in male CHCs as a product of interacting with different female strains, we exposed Z53 and DGRP‐882 males to females from their own and opposite strains, while keeping additional virgin males with no contact as controls. After allowing courtship for 10 min we separated the pairs and placed males in individual vials for 30 min followed by immediate CHC extraction, hoping that males would be exposed to females but would avoid physical contact via copulation. Within the 10 min, however, all of the flies had copulated except DGRP‐882 males paired with Z53 females. We did not find, however, previously identified female specific CHCs on the males, confirming that any changes to the males were not the result of female CHCs rubbing off of males (Khallaf, Cui, et al., 2020). Quantification of CHCs is described in the Supporting Information.

### 2.6 Statistical analyses

#### 2.6.1 Summarizing courtship behaviour

To accurately estimate copulation latency and the percentage of time spent on each behaviour we needed to determine when courtship is initiated. We determined courtship initiation in two ways: the time when the first non ‘separate’ behaviour took place or the time after three consecutive non ‘separate’ behaviours took place. The rationale behind the second method was to account for observations where males and females were close and scored as ‘engaging’ but courtship was broken off and there was a long gap before courtship was reinitiated. For the majority of the cases the difference between courtship initiation based on the two methods was minimal (mean = 7.4 s) and did not influence the overall estimate of copulation latency. For a few observations there was up to 4 min difference (max = 262.318 s) and re‐evaluating these outliers it was clear that the first ‘engaging’ behaviour was not courtship initiation. Given this comparison, we used the second method as it was more accurate. The percentage of time spent in a particular behaviour was calculated as the number of timepoints scored as this behaviour, divided by the total number of timepoints recorded until copulation occurred. We determined that courtship was similar across recording periods and determined when copulation failure was a result of males not initiating courtship (Supporting Information).

#### 2.6.2 Mating rejection in long‐term experiments

After analysing videos we observed that there were several female × male combinations that failed to copulate. We expected Z53 to reject non‐African males; however, there were several combinations that we did not *a priori* expect to fail, including Z53 females × LZ21 males and DGRP‐882 females with Z30 and LZ21 males. To determine if this was a function of our short recording time we set up single‐pair mattings between these genotypes and measured the time until progeny were first seen over a 16‐day period (Supporting Information).

#### 2.6.3 Plasticity analysis

To determine if male trait values for both behavioural traits and CHCs changed with respect to which female genotype a male was courting, we looked for evidence of consistent phenotypic plasticity across genotypes. We evaluated models that included male and female strain and their interaction on each trait. The male strain effect would capture differences between males. The female strain effect would indicate plasticity if males, on average, exhibited different trait values when interacting with different females. This pattern would graphically appear as parallel reaction norms (Roff, 1997). We included the interaction effect into our models because this would demonstrate that males differed in their effects and did not have consistent plasticity. To test these effects we used aligned ranks transformation (ART) ANOVAs using the ARtools package in R (Kay & Wobbrock, 2019). Details of these models and rationale for using them are included in the Supporting Information.

We also tested for plasticity in how the courtship behavioural sequence was carried out by males when interacting with different females. We quantified this difference using transitions between behavioural states. Behavioural sequences can be viewed as Markov chains, and analysing transition matrices in this Markov chain framework can determine differences in a behaviour sequence in courtship behaviour (Gaertner et al., 2015; Markow, 1987). First we constructed a transition matrix by summing all of the transitions across all trials for a specific female strain × male strain combination. Then we tested whether the transition matrix for a given male strain differed when interacting with the different female strains, using a homogeneity test from the Markov package in R (Spedicato, 2017). If these matrices were not homogeneous we could infer that the overall transition rates were different but not which particular transitions were responsible for this pattern. To determine if there were consistent transition differences we looked for plasticity of individual transitions after a variable reduction process using the VSURF package in R (Genuer et al., 2019).

#### 2.6.4 Detecting selection acting on male courtship traits

To determine if directional selection was acting on particular male traits and whether it was acting in the same direction across the two female strains, we used quantile regression to determine relationships between standardized male trait and relative fitness. We calculated standardized male trait values by subtracting the mean from each observation and dividing by the standard deviation, which resulted in a distribution with mean of 0 and standard deviation of 1 (Jones, 2009). Relative fitness was the courtship latency divided by the mean courtship latency, resulting in the mean of 1 but variance unaltered (Jones, 2009). The slope of the relationship between relative fitness and standardized trait value represents the selection gradient acting on this trait (Jones, 2009). We chose this analysis framework for two main reasons. First, using standardized male trait values allowed us to compare the strength of selection across traits and between female strains, since the distributions of males traits were not the same because of plasticity and some males not being represented in both samples. Second, using relative fitness allowed positive regression coefficients to represent positive selection.

To reduce the number of tests conducted, we used variable reduction using the VSURF package in R (Genuer et al., 2019) to determine which traits would be used to estimate selection gradients. We conducted variable selection for all male courtship and CHC traits simultaneously. For this classification problem relative fitness could be explained by the standardized male trait. We completed this process for both female strains separately, but then analysed all traits identified for both female strains.

For the linear selection analysis we used quantile regression to condition our regression model on the median rather than the mean, which is useful for non‐normally distributed data and robust to the presence of outliers (Koenker, 2005). We fit models for each female strain separately and calculated 95% confidence intervals based on the median using the package quantreg in R (Koenker, 2019). We evaluated selection gradients in the female strains separately given the differences in trait distributions caused by male plasticity described above. For these regressions the sample size was *n* = 13 for the DGRP‐882 female genotype (mattings with the male genotypes Z30 and LZ21 were excluded) and *n* = 13 for the Z53 female genotype (Canton‐S and LZ21 were excluded). We did not include variation within each genotype in the model as means were used instead of all trails. Because we did not measure CHCs for each male used in the courtship trials, we used the mean to represent the CHCs of male genotypes. To be consistent we correspondingly used the mean for male behavioural traits, which was also necessary given the differences in sample sizes of successful trials. The identity of males and female strain or species can be included in models to control for conspecific vs heterospecific effects (Boughman et al., 2005; Garlovsky et al., 2020), but we did not include this in our model because we can not *a priori* classify males into Z‐ or M‐type lineages based on collection location, and these lineage designations may not be biologically meaningful (see Section 1). Collection location does not predict courtship phenotype, as African genotypes can show phenotypes similar to non‐African genotypes (Coyne et al., 1999; Grillet et al., 2012; Yukilevich & True, 2008), and there are also large differences in phenotypes within African strains (see Section 3).

We initially focused on copulation latency because most male strains had relatively high success rates of mating. To incorporate some of the variability in mating success, we used the mating index described in Scott et al. (2011). This allowed us to include male DGRP‐882 courting Z53 females. While this combination did not mate, we could assign it the maximum time observed (which is an underestimate) but then weight it by the proportion of trials that mated. To directly estimate whether traits contributed to differential mating success we analysed the proportion of successful mattings using binomial regression (see below). Disentangling the effects of female preference and male courtship defects, and their interaction, on copulation success can be difficult. While *D*. *melanogaster* males generally court vigorously, including with other species (Seeholzer et al., 2018) and even inanimate objects (Kohatsu & Yamamoto, 2015), some isogenic genotypes exhibit significant inter‐individual variation in courtship intensity. For example, even within very favourable experimental conditions, with some males fail to court (Reza et al., 2013). Because male preference does not contribute to reproductive isolation between these strains (Coyne & Elwyn, 2006; Hollocher et al., 1997), our analysis of mating success and fitness is comprehensive, and would not be influenced by this potential source of copulation latency and mating success.

#### 2.6.5 Correlations between courtship traits and cuticular hydrocarbons

To determine which combinations of traits cluster and whether there is a common courtship suite for Southern Africa, we used principal component analysis (PCA) on the combined male courtship and behaviour data to identify strongly correlated traits. We focused on trials when males were courting Z53 females because males exhibited more variation in courtship behaviours when courting these females.

## 3 RESULTS

### 3.1 Courtship of African and non‐African males is significantly different

We quantified the behaviour of a standard *D*. *melanogaster* lab strain (DGRP‐882), a typical Southern African genotype (Z53), and one *D*. *simulans* genotype (SA22) to identify similarities and differences among them. We identified several behaviours in the Z53 strain that did not occur in the DGRP‐882 strain, but do occur in other species of the *melanogaster* subgroup. This included scissoring, where males open and close their wings rapidly (Video S2), similar to scissoring found in *D*. *simulans* (as defined in Cobb et al., 1985). We also observed circling, where males move around the female in an arc while vibrating or scissoring at the same time (Cobb et al., 1985; Video S1). In addition, the overall time spent on behaviours common between the two *D*. *melanogaster* strains was significantly different. Specifically, the Z53 strain spent less time singing compared to the DGRP‐882 strain, but more time singing than *D*. *simulans* (Figure S1), consistent with previous observations that African males sing less than non‐African males (Moran, 2006). For circling, we did not observe this behaviour in the non‐African strain but there was ample variation across African strains.

### 3.2 Geographic strains are reproductively isolated

The nature and degree of reproductive isolation is well documented in this system; however, we observed interesting and unexpected patterns that lead us to subsequently compare patterns of isolation among several genotype combinations. Despite active male courting, Z53 females strongly rejected both DGRP‐882 and Canton‐S males, consistent with previous reports (Wu et al., 1995). This rejection persisted much longer than the limited time we video recorded. To expand our ability to assay isolation between strains, we used progeny production over 16 days as a proxy for mating success. Comparing control mattings between Z53 females × Z53 males with mattings between Z53 females and several other strains, we saw that larvae took significantly longer to appear in crosses between Z53 females and DGRP‐882 and Canton‐S males as well as for ZH33 males (Figure S2A). Due to the small sample sizes and crossing survival curves, we could not determine if these male strains were significantly different from each other, but, qualitatively, fewer crosses produced larvae when DGRP‐882 and Canton‐S were paired with Z53 females (Figure S2A).

Based on the literature we did not expect reproductive isolation between non‐African females and African males (Coyne & Elwyn, 2006; Wu et al., 1995). Nevertheless, some male strains did not mate within the 30‐min video recordings, so we again assayed for progeny production. For crosses with DGRP‐882 females we used ZH33 males as our baseline because our sample size was too low for DGRP‐882 × DGRP‐882 crosses (many females or males died during the course of the experiment and these observations were not used). We reasoned that ZH33 was an appropriate substitute because they quickly produced both eggs and larvae when crossed with DGRP‐882 females, indicating that they mated within the first 24 h (Figure S2B). Compared to ZH33 males, both LZ21 and Z30 males took significantly longer to produce larvae and in fact very few mattings were successful over the course of the 16‐day experiment (Figure S2B). This level of reproductive isolation was similar in magnitude to the Z53 female × DGRP‐882 and Canton‐S male crosses. We conclude that isolation is not fully asymmetric and that males contain significant variation in mating success.

### 3.3 Male courtship behaviour is plastic

**FIGURE 1:**
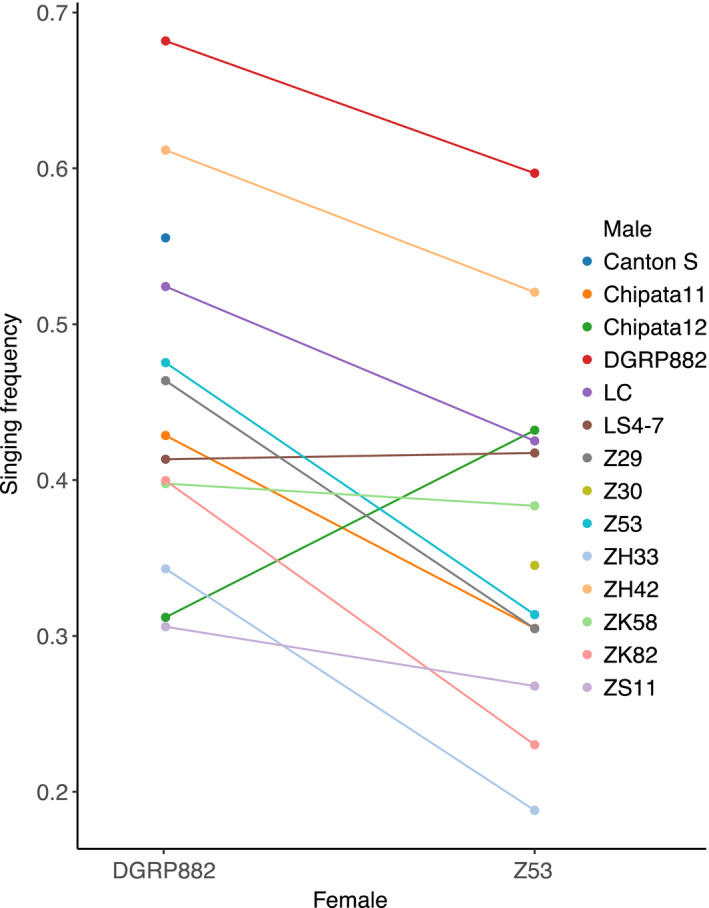
Male courtship singing frequency is female‐strain‐dependent. Male strains consistently decreased singing frequency when presented to a Z53 female, compared with being presented to a DGRP‐882 female. Each point represents the mean singing frequency when presented to a female of either type. Male strains are coloured alphabetically. Points that occur only in Z53 or DGRP‐882 female backgrounds indicate that males did not copulate with the other strain. This includes Canton‐S males only copulating with DGRP‐882 females and Z30 males only copulating with Z53 females. DGRP‐882 male behaviour was analysed when courting Z53 females even though they did not copulate

Having found significant differences between the courtship of Z53 and DGRP‐882 males, we next tested whether male courtship is a static or plastic trait. Using a panel of male strains we quantified the differences in behaviours when males interacted with either the Z53 or DGRP‐882 female strain. For the majority of the male strains, the time spent carrying out specific behavioural traits was female‐genotype‐dependent. The two traits that showed consistent plasticity were attempted‐copulation and singing (Table 1). The strongest signal was for singing, where males consistently spent more time singing when courting the DGRP‐882 female strain (Figure 1). Engaging and circling also had significant genotype effects but there was not a consistent pattern of plasticity across genotypes (Table 1).

**TABLE 1:** Male courtship behavioural traits are female‐strain‐dependent

Trait	Male‐genotype effect		Female‐genotype effect		Interaction	
	*F* value	*p*‐value	*F* value	*p*‐value	*F* value	*p*‐value
Engaging	**5.05**	**<0.0001**	0.031	0.8598	**2.264**	**0.0211**
Attempting	**3.88**	**0.0002**	**28.844**	**<0.0001**	1.759	0.0808
Singing	**7.93**	**<0.0001**	**15.826**	**0.0001**	1.016	0.4363
Circling	**3.92**	**0.0002**	**29.846**	**<0.0001**	**4.073**	**0.0001**
Scissoring	**8.49**	**<0.0001**	3.743	0.0563	1.596	0.1214

Note

The male strain effect captures differences between male strains in a courtship trait. The female strain effect indicates plasticity. An interaction effect indicates that changes in male strain behaviour is not consistently parallel across strains. Significance was determined using ART ANOVA and significant effects (*p* < 0.05) are reported in bold.

### 3.4 Courtship plasticity changes the behavioural sequence

Given that the time spent on behaviours was plastic, this could affect the behavioural sequence and transitions between behaviours. We therefore compared transition matrices for male strains interacting separately with the two female strains. The transition matrix was estimated from all transitions for a given male strain so is not biased by courtships that were overly short or long. Seven out of the 12 male strains had significantly different transition matrices (Table S1). The transitions that contributed to this plasticity were singing‐attempted‐copulation and engaging‐singing (Table S2). When interacting with Z53 females the male strains transitioned more from singing to attempted‐copulation and transitioned less from engaging to singing (Figure S3). This reflects that males were singing less in this interaction, consistent with our plasticity result for singing. The transition between singing‐scissoring had significant differences between male strains, but was not female‐strain‐dependent (Table S2).

### 3.5 Male cuticular hydrocarbons are plastic

We next tested the hypothesis that males change their CHCs upon exposure to females by quantifying CHCs in Z53 and DGRP‐882 males that had either been kept virgin or were exposed to Z53 and DGRP‐882 females. After variable reduction we were left with five compounds from the original 21 that we had identified, four of which had significant strain and treatment effects (Table S3). The compounds 5‐pentacosene (5‐C25) and 9‐pentacosene (9‐C25) showed similar patterns, with large differences between DGRP‐882 and Z53 males in all treatments (Figure S4). Z53 males had reductions in 5‐C25 when exposed to either female genotype (Figure S4). The other two compounds had changes that depended on both the male and female strain. Males reduced their amount of the compound 7‐tricosene (7‐C23) when they were exposed to females of the opposite type. For 2‐methyl‐triacontane (2‐Me‐C30), DGRP‐882 males increased their amount of this compound with DGRP‐882 females, but Z53 males decreased this compound with DGRP‐882 females (Figure S4). The plastic changes may contribute to selection on CHCs in terms of mating success (see below).

### 3.6 Divergent selection acts on courtship traits

**FIGURE 2:**
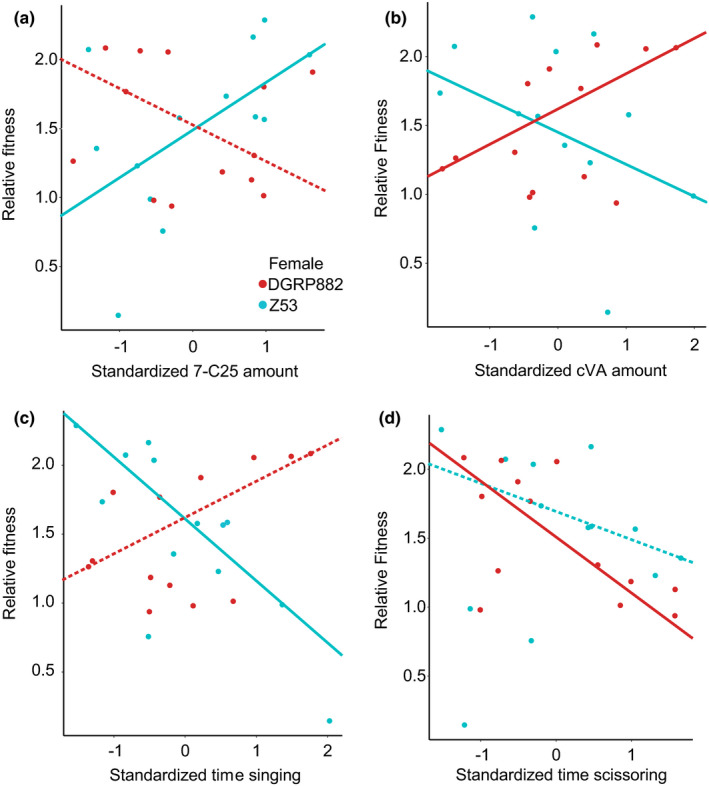
Directional selection on both male cuticular hydrocarbons (CHCs) and courtship traits is female‐genotype‐dependent. (a) Z53 females exert significant positive selection on the cuticular hydrocarbon 7‐pentacosene (7‐C25) and DGRP‐882 females show no significant selection gradient. (b) Selection on the compound cVA is divergent with opposite selection gradients between the female strains. (c) Z53 females do not respond to? singing and exert selection against this trait while no selection is estimated from the DGRP‐882 female strain. (d) DGRP‐882 females exert negative selection on this trait, while no significant relationship was found for Z53 females and this trait. Each point in the figures represents the mean value for each male strain when presented to a female of a particular strain, which was the value used in the statistical model. The regression lines were produced using quantile regression, with solid lines having a confidence interval different than zero, dashed lines having a confidence interval that contained zero

To test for directional selection on courtship traits in our two female strains, and divergent selection between these strains, we used quantile regression to determine the relationships among the seven CHC traits and two behavioural traits and relative fitness. We also determined if these traits increased the probability of mating success using binomial regression (Table 2). For linear selection gradients the CHC cVA was the only trait that showed divergent selection between the two strains (Table 2; Figure 2). In the binomial regression both n‐C21 and singing had divergent effects on the probability of successful copulation. The majority of traits had effects in only a single female strain and most showed effects on both relative fitness estimated through copulation latency, and the probability of copulation (Table 2; Figure 2). Overall, this resulted in different suites of traits experiencing both positive and negative selection in these two strains.

**TABLE 2:** Selection gradient coefficients and binomial regression coefficients for each trait identified through variable selection

Trait	Selection gradient Z53 F		Selection gradient DGRP‐882 F		Binomial regression Z53		Binomial regression DGRP‐882	
	Lower CI	Upper CI	Lower CI	Upper CI	Lower CI	Upper CH	Lower CI	Upper CI
n‐C21	−0.88	0.03	−0.31	0.70	**−1.25**	**−0.22**	**0.09**	**1.17**
n‐C22	−0.84	0.43	−0.27	0.54	*−0.89*	*0.01*	−0.16	0.76
n‐C24	−0.55	0.13	**−0.38**	**−0.29**	−0.07	0.85	**−1.02**	**−0.10**
7‐C25	**0.02**	**0.96**	−0.48	0.21	**0.02**	**0.97**	−0.43	0.42
2‐Me‐C26	−0.27	0.85	**−0.46**	**−0.19**	−0.23	0.70	**−1.21**	**−0.20**
7‐C23	−0.32	0.34	−0.24	0.54	***−0.87***	***0.05***	−0.35	0.50
cVA	**−0.42**	**−0.01**	**0.02**	**0.38**	−0.71	0.20	**0.11**	**1.06**
Singing	**−0.11**	**−0.71**	−0.14	0.45	**−1.08**	**−0.07**	**0.44**	**1.54**
Scissoring	−0.37	0.37	**−0.49**	**−0.24**	−0.12	0.80	***−0.86***	***0.02***

Note

We ran a separate regression for each female strain using relative fitness and standardized trait values to make each analysis comparable and report 95% confidence intervals. Intervals that are positive suggest a significant positive selection gradient. The binomial regression used the same trait data but the number of successful and unsuccessful trails as the response variable. Bold indicates a confidence interval not overlapping zero. The italics correspond to a coefficient that had *p* < 0.1 but confidence intervals that overlapped zero.

The CHC cVA and singing behaviour have been intensively studied in non‐African populations and are thought to be key male mating traits (Grillet et al., 2006; Kurtovic et al., 2007; Scott et al., 2011; Talyn & Dowse, 2004). Interestingly these are both under negative selection in the Z53 strain (cVA linear *β* 95% CI = (−0.42, −0.01); singing linear *β* 95% CI = (−0.11, −0.71)). Non‐African strains are thought to mate indiscriminately in both choice and no choice tests. Nevertheless, we found traits under negative selection in the DGRP‐882 female strain (Table 2). The only trait that we could identify as under positive selection in the Z53 strain was the CHC 7‐pentacosene (7‐pentacosene linear *β* 95% CI = (0.02, 0.96)). Overall, we detected more significant negative selection gradients than positive selection gradients, which may reflect the distribution of traits in our male strains, or a bias in this type of selection gradient estimation.

### 3.7 Variation in courtship traits results in discrete clusters of male genotypes

**FIGURE 3:**
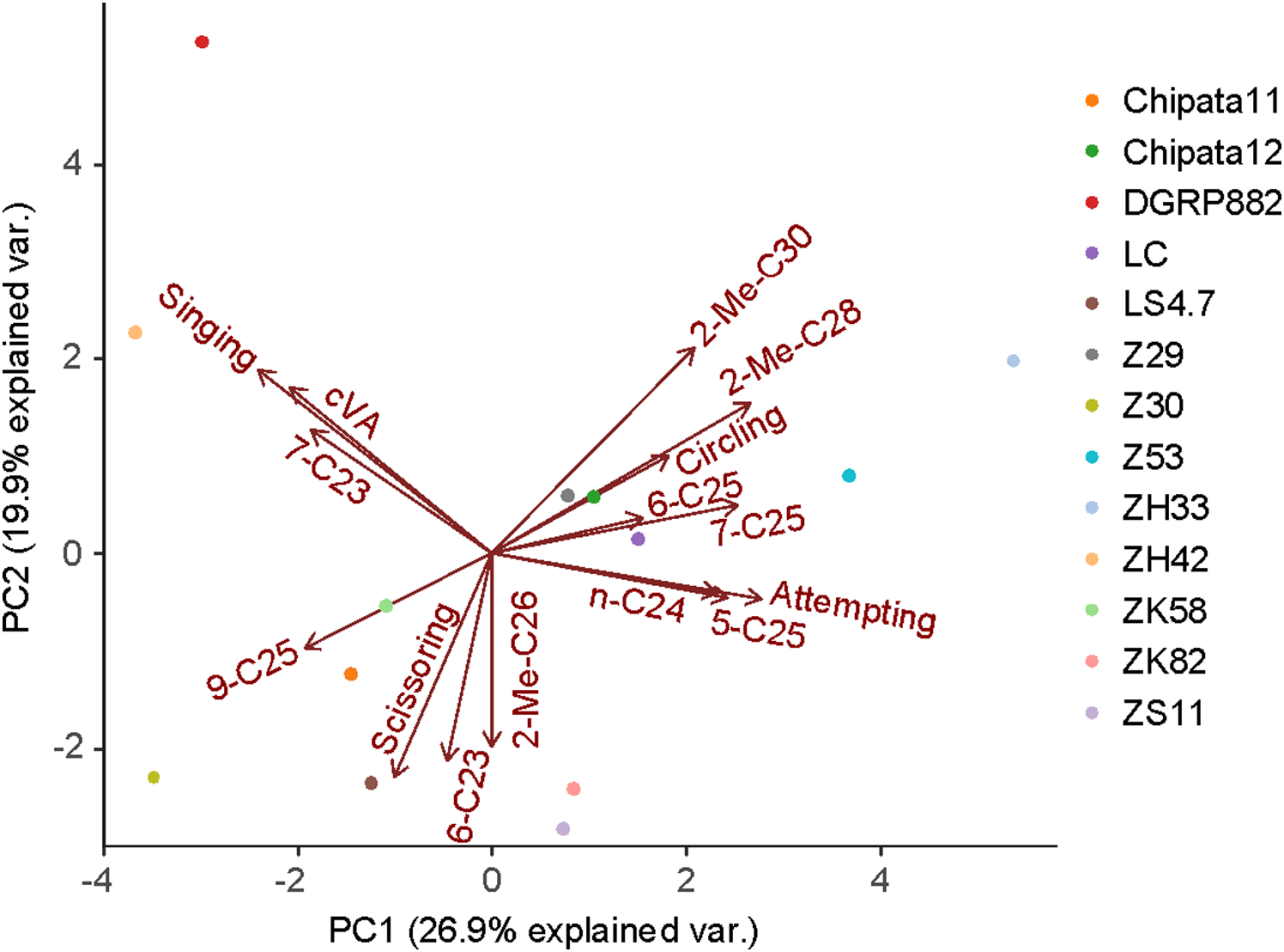
The combination of male cuticular hydrocarbons and behaviours that characterizes distinct male courtship phenotypes. The combination of singing behaviour and the cuticular hydrocarbons cVA and 7‐tricosene (7‐C23) defines one cluster that contains the non‐African male DGRP‐882. The African strains fall into two broad clusters defined by unique behaviour and CHC combinations. Each point represents the average value for a given male strain with behavioural data coming from interactions with Z53 females. Each arrow represents one main variable used to construct the principal components, with its length representing the loading on a particular principal component axis

Given the behavioural trait differences between the male strains and their impacts on mating success, we wanted to determine how the variation in courtship traits and CHC covaried among our male strains, in order to examine variation in courtship strategies in African males. To do this we determined the relationship between male CHCs and male behavioural traits when courting Z53 females using principal component (PC) analysis. While strains were spread across the PC space, there was clustering based on the loadings of specific combinations of behaviours and CHCs (Figure 3). For example, one cluster included ZH42 and DGRP‐882 and the loadings that contributed were Singing, cVA and 7‐C23. In the opposite direction in PC space, Z53 and a few other African strains clustered with contributing loadings including Attempted‐Copulation and C25 isomers. The last main cluster included Z30, LS4‐7, and other strains that were mostly separated by the Scissoring behaviour and additional CHC compounds. Overall, the different clusters suggest there is not a single courtship and CHC combination that is most common in these African strains.

## 4 DISCUSSION

While sexual selection appears to be an important driver of speciation based on macroevolutionary patterns, connecting contemporary divergent sexual selection to reproductive isolation has been challenging. Towards this end, we leveraged *D*. *melanogaster* to quantify selection on relevant male courtship traits for females from two geographically isolated female strains with whom they show different patterns of mating success. We comprehensively documented differences in male behaviour and cuticular hydrocarbons (CHCs) between the African and non‐African strains that allowed us to test for selection on these suites of traits, though more experiments are needed to demonstrate a direct and specific role for these traits in reproductive isolation and how consistent these selection gradients are across more female strains. Together our results demonstrate that ample variation exists in courtship traits between geographic strains of *D*. *melanogaster* and that this might be maintained by both male behavioural plasticity and differences in female preference for these traits between geographic locations.

Based on previous knowledge of *D. melanogaster* lab strains, we could make *a priori* predictions about which traits might be under positive selection in the DGRP‐882 strain. We confirmed that the time spent singing and the amount of the hydrocarbon cVA are under positive selection in this strain. We then showed that these traits are under negative selection in the Z53 strain, where increasing time spent singing or increased amounts of cVA decreased relative fitness (Figure 2). Interestingly, even though non‐African strains are thought to mate indiscriminately, we identified traits under negative selection by DGRP‐882 females. CHCs contribute to reproductive isolation in this system (Coyne et al., 1999; Grillet et al., 2012) and we identified one major compound under positive selection by Z53 females. For these experiments we used a single representative strain to represent females of each lineage. While speciation studies in *Drosophila* and other organisms vary in the number of strains used to represent a lineage (Jaenike et al., 2006; Matute, 2010; Patterson & Stone, 1952), in this scenario of early divergence, it will be important to determine if this selective pressure is consistent across multiple female strains. While previous research using African strains suggest that they all have similar rejection behaviours of non‐African strains (Hollocher et al., 1997; Moran, 2006; Wu et al., 1995), the level we are investigating is within population differences. Determining if there is variation within populations will be especially important now that African populations are not considered to be a single Z‐type lineage (Coughlan et al., 2021). Females in Africa that reject non‐African males might have different trait preferences, while maintaining the same level of reproductive isolation outside of their geographic origin. This could explain in part, why there is so much behavioural and cuticular hydrocarbon variation segregating in Africa (Figure 3) Regardless of the limited number of female strains used, our results clearly demonstrate that *D*. *melanogaster* strains are not homogenous in either male courtship behaviour or in the traits that females are using when making mating decisions.

Female mate choice is often correlated with the presence of a conspicuous fixed difference between species (Coyne & Orr, 2004; Mckinnon & Rundle, 2002; Qvarnstrom et al., 2010), but in young divergent lineages, trait variation may segregate between populations (Hendry et al., 2009; Khallaf, Auer, et al., 2020; Mallet, 2008; Merot et al., 2017). As a result, both quantitative differences and the presence or absence of traits could shape reproductive isolation. This difference in continuous vs discrete traits also influences how reproductive isolation and sexual selection are measured. Pre‐mating reproductive isolation is often estimated by the number of successful mattings compared to unsuccessful mattings (Coyne & Orr, 1989). Whereas for sexual selection trait values are correlated with a continuous variable that captures fitness because it is assumed that mattings within species will generally be successful (Brooks & Endler, 2001; Callander et al., 2012; Hill, 1991; Oh & Shaw, 2013; Rebarm et al., 2009; Steiger & Stokl, 2014). Since the goal of this experiment was to estimate sexual selection, we relied on copulation latency and mating success as a proxy for fitness. Using both metrics to estimate sexual selection captures the complexities in fitness. This includes female mate choice, male courtship intensity and vigour, and feedback between the sexes (Coyne & Elwyn, 2006; Moran, 2006). These effects cannot be disentangled and sometimes does reflect mating deficiencies of individuals rather than mating interactions (Reza et al., 2013; and see Section 2 and Supporting Information). For young lineages it will be important to determine how mating success is related to other measures of fitness in the context of sexual selection.

We identified examples of divergent selection between female strains by leveraging the large phenotypic variation for both courtship behaviour and CHCs among the African strains in our experiment. We focused on strains from Southern Africa to maximize trait diversity, but could not *a priori* predict how differentiated these strains would be from non‐African strains. Given the recent and historical gene flow of European ancestry back into Africa (Pool et al., 2012) one might expect some of the courtship variation in Southern Africa to be due to this gene flow, with males phenotypically appearing either similar to non‐African males or intermediate in phenotype. Indeed, we saw this with ZH42, which was similar in both behaviour and CHCs to the non‐African genotypes, but we also observed ample variation segregating within African populations (Figure 3). Interestingly, the traits under selection that we identified generally occur in both lineages, so if these traits contribute to reproductive isolation it will more likely be due to the time spent on a behaviour or the quantity of a CHC rather than presence/absence of a specific male trait.

We discovered that some African strains carry out two behaviours, scissoring and circling, that we did not see in the two non‐African strains. Circling was not under selection for either female strain, but scissoring had a more interesting pattern. Scissoring has previously been observed in *D*. *melanogaster* strains from the Bahamas and Caribbean that contain African ancestry, but it was a minor part of their courtship (Yukilevich & True, 2008). We found African strains display scissoring behaviour at a higher frequency than non‐African strains. Increased time spent scissoring increased the time it took for copulation to occur and decreased the probability of copulation for DGRP‐882. In fact, we saw very strong reproductive isolation between DGRP‐882 females and African males from the strain that displayed the most scissoring. This level of reproductive isolation was as strong as the isolation that is typical between Z53 females and non‐African males (Figure S2). Scissoring thus provides an interesting example where a lineage‐specific trait may contribute to reproductive isolation and shape female mate preference, but is not under divergent linear selection.

Both sexual selection and environmental selection can shape patterns of CHC divergence (Chung et al., 2014; Greenberg et al., 2003; Higgie et al., 2000), and are examples of magic traits, contributing to both ecological divergence and reproductive isolation (Servedio et al., 2011). CHCs strongly contribute to reproductive isolation in newly diverged lineages and often these differences are quantitative (Chung et al., 2014; Khallaf, Auer, et al., 2020; Seeholzer et al., 2018; Veltsos et al., 2012). By demonstrating divergent selection acting on CHCs we are able to identify compounds that could contribute to reproductive isolation, and follow‐up studies can test this by manipulating these compounds and measuring reproductive isolation. Previous studies did not determine which CHCs are under positive selection in African lineages. This was likely a product of the phenotypic distribution of the strains in those studies and the focus on compounds that are typically in high quantities in non‐African strains, specifically isomers of tricosene (Grillet et al., 2012). For the intensively studied compound 7‐tricosene (7‐C23) we recapitulated previous reports that it decreases the likelihood of mating success for African female strains (Grillet et al., 2012), but could not detect positive selection for the non‐African genotype. One possibility is that all genotypes had the minimum amount of 7‐C23 that would ensure acceptance by the DGRP‐882 females. If there is a threshold for mating acceptance based on this compound, it would be consistent with the idea that non‐African females mate indiscriminately (Wu et al., 1995).

Another mechanism that could contribute to the asymmetry of reproductive isolation in this system is that male courtship is female‐strain‐dependent. Male courtship is described in seemingly contradictory terms, sometimes as a stereotypical sequence (Cobb et al., 1985; Gaertner et al., 2015), but other times as plastic (Arbuthnott et al., 2017; Dukas & Dukas, 2012; Filice et al., 2020; Marie‐Orleach et al., 2019, 2020). This discrepancy might come from the fact that plasticity is often quantified in terms of courtship effort and intensity, rather than the behavioural sequence. We identified behaviours that were consistently plastic across strains and resulted in changes to the fundamental behavioural sequence (Figure 1). We also detected significant plasticity for some CHCs in *D*. *melanogaster*, which can be further explored in future studies. Plasticity that is female‐strain‐dependent has the potential to impact how we view sexual selection, by creating scenarios where males can maintain fitness in the presence of variation in female mate preference. Selection can maintain plasticity in recently diverged lineages where males come into contact with multiple female genotypes and gain fitness by adjusting their courtship displays. Asymmetrical reproductive isolation, which is especially common in *Drosophila* (Yukilevich, 2012) and other systems where both females and males contribute to mate choice, might also reflect plasticity. For example, even though non‐African males, such as DGRP‐882, reduced the time spent singing when they courted Z53 females, they still spent significantly more time singing than the African strains and ultimately failed to mate with Z53 females. Female‐strain‐dependent courtship plasticity suggests that males require specific female cues for successful courtship (Barker, 1967; Cobb & Jallon, 1990; Seeholzer et al., 2018). CHCs can both stimulate and inhibit mating (Ejima et al., 2007; Ferveur, 2005; Grillet et al., 2006; Khallaf, Auer, et al., 2020; Khallaf, Cui, et al., 2020), and a strong difference exists in the female CHCs of African and non‐African females (Coyne et al., 1999; Dallerac et al., 2000). The ability of males to change their courtship might alter courtship in subsequent mating interactions, which are typically not considered in the study of speciation.

The interaction between plasticity and learning can have important impacts on the strength of reproductive isolation and outcomes for speciation (Kujtan & Dukas, 2009; Verzijden et al., 2012). Successful mating can create positive associative learning (Ejima et al., 2007; Griffith & Ejima, 2009; Koemans et al., 2017; Zer‐Krispil et al., 2018). If males adopt a successful courting strategy as a result of this conditioned learning they may show less plasticity in subsequent mating bouts (Dukas & Dukas, 2012). If instead, plasticity is maintained across subsequent mating encounters, then gene flow would continue and reproductive isolation would not evolve (Kirkpatrick & Nuismer, 2004; Pfennig et al., 2010). Plasticity within a species could therefore maintain variation in male courtship and female preference would persist in populations. This segregating variation could potentially facilitate rapid speciation, but only after geographic separation (Castillo & Delph, 2016; Mendelson et al., 2014).

We identified large differences in male behaviours and CHCs among African strains, such that we could not define a single behavioural trait and CHC combination that classifies African vs non‐African males. This intriguing result suggests that there are segregating preferences in these populations, which could be a product of complex genetic structure within Southern Africa (Begun & Aquadro, 1993; Coughlan et al., 2021; Dieringer et al., 2005; Pool et al., 2012). This variation may have facilitated the rapid divergence of the non‐African lineages from African ancestors when these lineages migrated out of Africa. This divergence has now created strong asymmetric reproductive isolation that is potentially maintained by ongoing divergent sexual selection. Future studies will need to manipulate trait values for the CHC and courtship traits we identified and quantify changes in premating reproductive isolation in order to make explicit connections between sexual selection and reproductive isolation. Quantifying the strength of selection across female genotypes will help to determine how consistent selection is in these geographic populations and whether there is local mate selection that could maintain variation within the larger context of behavioural isolation across *D*. *melanogaster* populations.

## CONFLICT OF INTERESTS

The author's declare no conflict of interest.

### PEER REVIEW

The peer review history for this article is available at https://publons.com/publon/10.1111/jeb.14007.

## Supporting information

Supplementary Material

Video S1

Video S2

## Data Availability

All data and R code used in this study are available through the Dryad Digital Repository https://doi.org/10.5061/dryad.vdncjsxx6.
